# Structural Properties of Chemically Functionalized Carbon Nanotube Thin Films ^†^

**DOI:** 10.3390/ma6062360

**Published:** 2013-06-10

**Authors:** George Trakakis, Dimitrios Tasis, John Parthenios, Costas Galiotis, Konstantinos Papagelis

**Affiliations:** 1 Institute of Chemical Engineering and High Temperature Processes, Foundation of Research and Technology Hellas, P.O. Box 1414, Patras GR-26504, Greece; E-Mails: trakakis@iceht.forth.gr (G.T.); dtassis@upatras.gr (D.T.); jparthen@iceht.forth.gr (J.P.); c.galiotis@iceht.forth.gr (C.G.); 2 Department of Materials Science, University of Patras, Patras GR-26504, Greece

**Keywords:** carbon nanotubes, buckypaper, oxidation, epoxidation, mechanical properties, electrical properties

## Abstract

Buckypapers are thin sheets of randomly entangled carbon nanotubes, which are highly porous networks. They are strong candidates for a number of applications, such as reinforcing materials for composites. In this work, buckypapers were produced from multiwall carbon nanotubes, pre-treated by two different chemical processes, either an oxidation or an epoxidation reaction. Properties, such as porosity, the mechanical and electrical response are investigated. It was found that the chemical pretreatment of carbon nanotubes strongly affects the structural properties of the buckypapers and, consecutively, their mechanical and electrical performance.

## 1. Introduction

Carbon nanotubes (CNTs) have become one of the main scientific subjects during the years after their discovery [1], due to their exceptional properties [2]. They have been studied either as isolated nanostructures or in the form of macroscopic assemblies, such as CNT papers and films [3,4], yarns [5], fibers [6], forests [7], *etc.* More specifically, CNT films, the so-called “buckypapers”, have been used as functional components in various applications, including catalyst supports [8], permeable membranes [9], actuators [10], capacitors [11], electrodes for fuel cells [12], electrical conductive components [13] and reinforcement in composite materials [14].

Buckypapers are thin membranes of randomly entangled CNTs, which form highly porous networks, mainly due to van der Waals interactions. The most common way to produce them is to disperse pristine or chemically modified nanotubes in a solvent medium [3,4] and, subsequently, filtrate the suspension through a microporous membrane. Recent studies have found that the structural, as well as the electrical properties of the formed CNT networks may be affected by various parameters, such as the chemical modification of the tubes, CNT length, diameter and number of walls, the degree of alignment of the carbon nanostructures and the solvent medium used for the dispersion of the nanotubes [3,15,16,17]. A number of different protocols for buckypaper fabrication have been reported, giving CNT papers with a variety of interesting properties [10,16,17,18,19,20,21,22,23,24,25,26]. This wide variation of the buckypaper properties necessitates the thoughtful selection of the parameters affecting the structure of the buckypaper, depending on the targeted application. For example, using a buckypaper as a permeable membrane demands a proper porosity distribution. On the other hand, its utilization as an electrically conductive component requires a high degree of interconnections between adjacent tubes.

In the area of structural materials, CNT networks are recognized as potential high performance filler for composites of enhanced mechanical properties [27,28,29,30,31]. In addition, buckypapers are strong candidates as reinforcing agents in laminated polymer composites structures [32,33]. The mechanical integrity of neat CNT buckypapers can be greatly enhanced by optimizing the packing quality of the carbon nanostructures during the filtration step, which leads to an increased number of interconnects between the adjacent tubes in the formed membrane. This may be achieved by preparing a stable CNT suspension containing individually dispersed tubes. On the other hand, precursor CNT suspensions containing both individual and bundled tubes may lead to the preparation of buckypapers with larger pore sizes, decreased number of interconnections and, eventually, lower mechanical integrity.

In this work, different types of buckypapers were produced by varying three processing parameters. These include the chemical pretreatment of the CNT material, the chemical environment in which the tubes were dispersed and the drying protocol during the final stage of the buckypaper formation. The aim is an in-depth understanding of the structure-properties correlation, to end up with a suitable buckypaper for a number of applications.

## 2. Results and Discussion

During the first stage of buckypaper formation, the starting CNT material was subjected to two different chemical modification approaches (epoxidation and oxidation) in order to succeed in the exfoliation of carbon nanostructures in solution. Moreover, the aforementioned modification schemes give rise to the covalent decoration of a graphitic surface with oxygen-containing functionalities. The presence of the latter seems to enhance the interactions between adjacent tubes in the formed buckypaper through hydrogen bonding forces, which collaborate with the existing van der Waals forces, due to the graphitic nature of the CNT surface. The strength of these interactions crucially affects the mechanical behavior of the buckypaper. Very strong interactions push the nanotubes to adhere strongly between each other, forming a structurally integrated buckypaper. On the contrary, weak CNT interactions lead to a more “loose” network.

Another crucial parameter that affects the structure of the buckypaper is the chemical affinity between the carbon nanostructures and the solvent medium used for its exfoliation in solution. The solvent molecule-nanotube interaction is the key factor, which finally governs the dispersion quality and, eventually, defines the packing density of entangled tubes in the buckypaper structure. If the nanotubes have good solubility in a specific solvent, then they are individually dispersed in solution, creating a very dense structure after the filtration of the CNT suspension. On the other hand, if the nanotubes are inadequately exfoliated, they form agglomerates, and the resulting buckypaper develops large pores. Besides the influence of solvent polarity, the final structure of the film can be strongly affected by the boiling point of the solvent, hence, the drying rate after the filtration process. If the solvent is evaporated comparatively rapidly, the nanotubes have no time for re-arrangement, and they remain in a more “foamy” state. To this goal, we used two media that possess variable polarity, as well as different boiling points (dichloromethane and water). In addition, we varied the processing conditions concerning the filtration step. Except for the regular filtration protocol, we tried to produce buckypapers in which the vacuum-assisted process is terminated slightly before all the solvent was filtrated and then performed drying of the wet buckypaper by applying a hot air supply. In this concept, the nanotubes are entangled in a more “loose” integrity (“Foamy” processing). Details of the fabrication protocols are given in the Experimental Section.

Another parameter is the oxidative nature of the chemical treatment. Oxidation reaction is considered a harsh condition process, leading to cutting of nanotubes into smaller tubes. The latter can pack better, forming a more dense structure.

**Figure 1:**
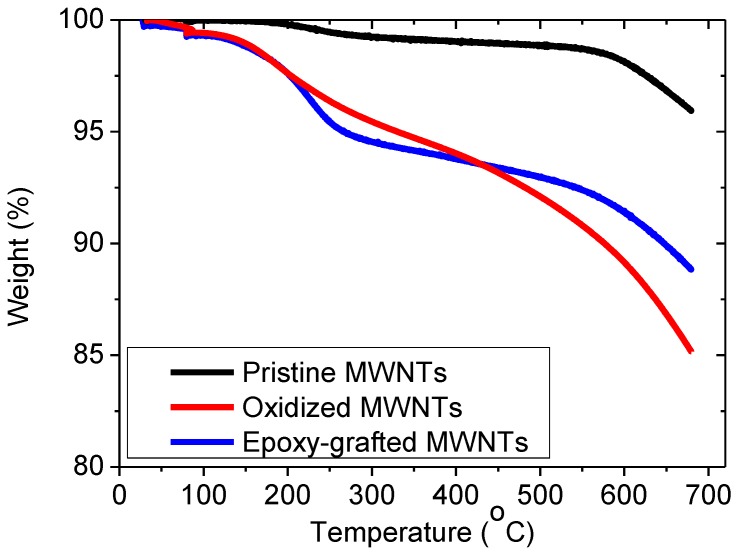
Thermogravimetric analysis of pristine, oxidized and epoxidized nanotubes.

Thermogravimetric analysis (TGA) was used to examine the grafting density of the oxygen containing groups onto the graphitic surface. These involve hydroxyl and carboxyl functionalities during oxidation reactions or epoxy ones during epoxidation. Figure 1 shows the weight loss of pristine, oxidized and epoxidized multi-wall carbon nanotubes (MWCNTs) as a function of temperature. As can be seen, there is a clear difference in the thermogravimetric curves of the chemically modified samples compared to untreated ones, which is due to the pyrolysis of the functional groups attached on the MWCNTs surface. It was calculated that the mass percentage of the functionalities in the oxidized and epoxidized material was about 11% and 8%, respectively.

**Figure 2:**
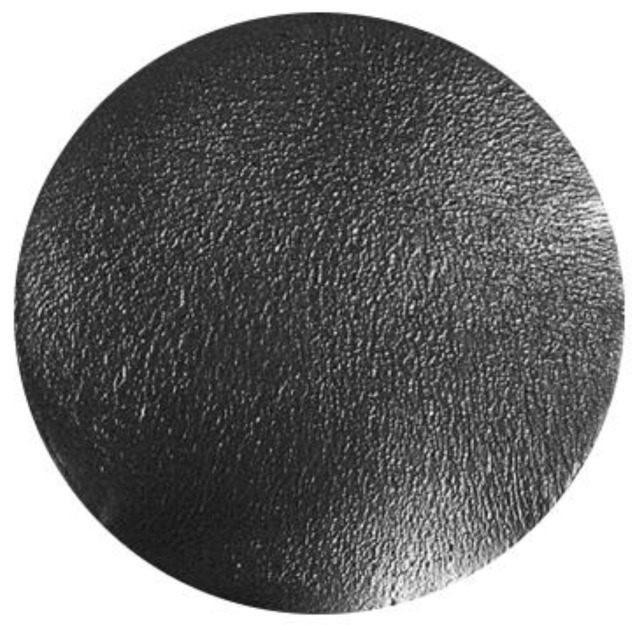
Optical image of a multi-wall carbon nanotube (MWCNT) buckypaper.

**Figure 3:**
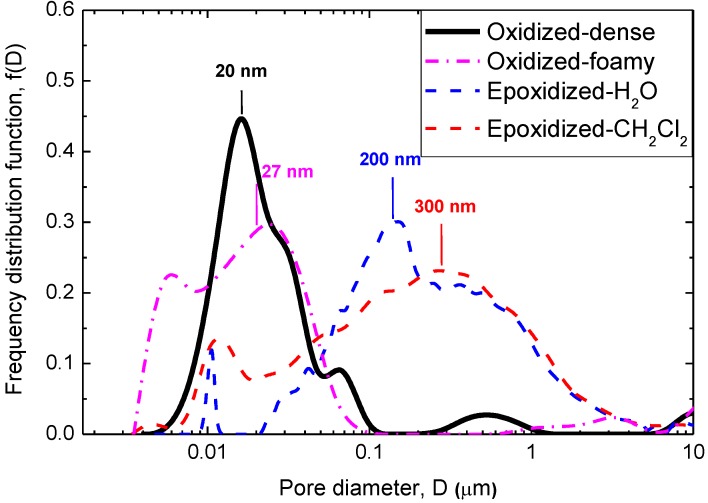
Pore size distribution from Hg intrusion curves of oxidized and epoxidized buckypapers.

A typical optical image of the produced buckypapers is shown in Figure 2. To examine the structure of the buckypapers, porosimetry measurements, as well as scanning electron microscopy (SEM) imaging were recorded. Figure 3 shows the pore size frequency distribution of the produced buckypapers, while Table 1 gives the extracted data from the porosimetry experiments. As it can be seen, both oxidized buckypapers in water exhibit very small pore sizes with a profile maximum at about 20 nm. By using the alternative drying protocol to create a more foamy structure, pore sizes of about 27 nm were achieved. It is noted that such pore sizes are unsuitable for the infusion of macromolecular chains within the free interstitial galleries of the CNT network. This fact makes the oxidized buckypapers unsuitable for use as a reinforcement material for nanocomposite production [14], as the unfilled pores can crucially affect the properties of the nanocomposites [34,35] On the contrary, epoxidized buckypapers both in water and dichloromethane exhibit larger pore sizes of a few hundreds of nanometers. The shift of the pore size distribution maxima towards higher values for epoxidized tubes could be attributed to their decreased dispersibility in both media.

**Table 1:** Data from porosimetry experiments. *V*_p_ is the intruded Hg volume per gram of material and *φ* is the % porosity.

Buckypaper	*V*_p_ (cm^3^/g)	*φ*
Oxidized-dense	0.6718	0.55
Oxidized-foamy	0.6467	0.63
Epoxidized-H_2_O	2.6698	0.85
Epoxidized-CH_2_Cl_2_	3.1470	0.88

**Figure 4:**
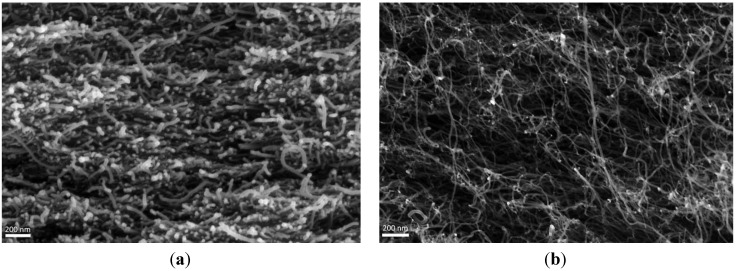
(**a**) SEM photo of an oxidized buckypaper; (**b**) SEM photo of an epoxidized buckypaper.

Analogous conclusions can be obtained optically from the SEM photos (Figure 4). A first note here is that the oxidized buckypapers present a very dense structure. The nanotubes are arranged very close to each other, leaving almost no empty spaces between them. The opposite picture is found in epoxidized buckypapers, namely, the average distance between the CNTs is somewhat higher, which is strongly supported by the porosimetry results (Figure 3). In both cases, a uniform morphology of the CNT network can be observed, while at the nanotube level, there appears to be a random orientation of the carbon nanostructures.

**Figure 5:**
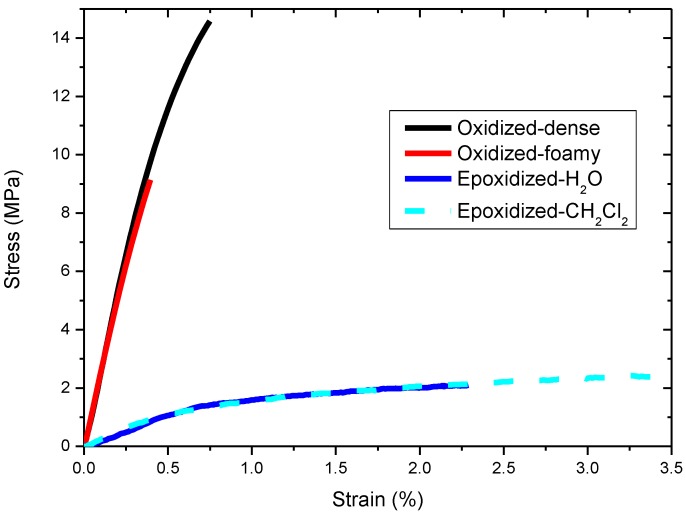
Tensile curves of the buckypapers.

The tensile properties of the studied buckypapers are shown in Figure 5 and Table 2. In Figure 5 are presented the stress-strain curves and, in Table 2, the tensile strength at fracture, the fracture strain and the elastic modulus. The oxidized buckypapers exhibit an enhanced fracture stress compared to the epoxidized ones. This is attributed to an increased number of CNT interconnects, giving rise to better structural integrity of the porous network and, hence, higher friction interactions between the tubes. More specifically, the oxidized-dense buckypaper has reached 14 MPa stress and 0.68% strain, while the Young modulus is 2.8 GPa. The oxidized-foamy film has a lower stress, due to its more “loose” structure. On the contrary, the epoxidized papers, due to their decreased bulk density, have fewer interconnections between the nanotubes, which means that lower friction forces are present. This is the reason why their mechanical properties are poor.

**Table 2:** Tensile data of the buckypapers: *σ* is the tensile strength at fracture, *ε* is the fracture strain and *E* is the Young modulus.

Buckypaper	*σ* (MPa)	*ε* (%)	*E* (GPa)
Oxidized-dense	14.03 ± 1.80	0.68 ± 0.10	2.83 ± 0.20
Oxidized-foamy	9.14 ± 2.00	0.44 ± 0.12	2.28 ± 0.25
Epoxidized-H_2_O	2.10 ± 0.12	2.35 ± 0.32	0.20 ± 0.02
Epoxidized-CH_2_Cl_2_	2.42 ± 0.16	3.42 ± 0.50	0.21 ± 0.04

An overview in the literature data, concerning the mechanical properties of CNT buckypapers, clearly shows a wide range of values for both tensile strength (2–80 MPa) and modulus (0.5–15 GPa) [3,4,16,36,37,38]. The variation at the measured values strongly demonstrates that the mechanical properties of CNT thin films are affected by a number of parameters, such as number of walls, length of carbon nanostructures, nature of chemical treatment, *etc.*

The DC electrical conductivity of the buckypapers is shown in Table 3. As it can be seen, the oxidized buckypapers exhibit higher electrical conductivity than the epoxidized ones. To interpret this behavior, it is necessary to understand the mechanism of the electrical conductivity in a carbon nanotube film. In general, the electrical conductivity of such films depends on two factors, namely, the conductivity of the nanotubes themselves and the ability of the electric carriers to tunnel between neighboring nanotubes. Concerning the first factor, we can reasonably consider that the electrical conductivity of oxidized and epoxidized nanotubes is very similar, as the nanotubes used in both oxidation and epoxidation treatments were the same, and the chemical treatment affected only the outer shell of these multi-wall nanotubes.

**Table 3:** DC electrical conductivity of the buckypapers.

Buckypaper	*σ* (S/cm)
Oxidized-dense	10.3
Oxidized-foamy	10.1
Epoxidized-H_2_O	7.1
Epoxidized-CH_2_Cl_2_	6.0

On the other hand, the second factor implies that the electric conductivity depends on the number of contact points or conductive channels between the nanotubes. As the network density increases, more conductive pathways for the charge carriers are available, yielding an increase in conduction with the film density. This explains the higher conductivity of the oxidized buckypapers compared to the epoxidized ones, since these films have a more dense structure. The same conclusion for the relation of film structure *vs.* electrical conductivity has also been extracted by Lyons *et al.* [39], who also reported electrical conductivity values in the range between 2.08 S/cm and 167 S/cm for single-wall CNT films.

The nanotube network architecture opens up significant opportunities for the use of buckypapers in a number of applications. For example, if an application employs the impregnation of the paper for a composite material production, the film must have large pores to allow the matrix to completely impregnate the nanotubes. Also, If an application demands high electrical conductivity or high elastic modulus and strength, the buckypaper should be as dense as possible, to increase the number of junctions and the friction between the tubes, respectively. Therefore, the application in which the buckypaper is going to be used defines the structural morphology the buckypaper should have.

## 3. Experimental Section

MWCNTs were grown by the chemical vapor deposition (CVD) method and were supplied by Nanocyl (Belgium). Potassium permanganate (KMnO_4_), sulfuric acid (H_2_SO_4_) and dichloromethane (CH_2_Cl_2_) were purchased by Sigma-Aldrich, whereas 3-chloroperoxybenzoic acid (70%–75%) was supplied by Acros Organics. The production of the buckypapers is composed of two steps; the modification of the nanotubes and the fabrication of the buckypaper.

Concerning the oxidation protocol [40], 2.6 g of multi-walled CNTs were dispersed in 260 mL of 0.5 M sulfuric acid by ultrasonic vibration for 5 min in a flask. The suspension was refluxed in an oil bath at 120 °C with magnetic stirring. Meanwhile, 25 g of KMnO_4_ was dissolved in 260 g of 0.5 M sulfuric acid, and this solution was added to the flask dropwise. The mixture was kept at 120 °C for 3 h. After that period, the resulting suspension was filtered, washed with hydrochloric acid and deionized water and then dried.

For the epoxidation reaction [41] of the CNT material, 7 g of 3-chloroperoxybenzoic acid were dissolved in 150 mL CH_2_Cl_2_. Then, 1.5 g of multi-walled CNTs were added, and the solution was stirred for about 20 h at room temperature. The reaction mixture was filtered through a 0.2 μm PTFE membrane filter and washed with excess CH_2_Cl_2_. The epoxidized CNT material was dispersed again in 150 mL CH_2_Cl_2_ by sonication, filtered and dried under vacuum at 80 °C.

Concerning the fabrication of multi-walled CNT buckypapers, consisting of the oxidized nanotubes, stable CNT suspensions in H_2_O or CH_2_Cl_2_ at a concentration of 1 mg mL^−1^ were prepared by tip sonication. The mass of the CNT material was 250 mg. These dispersions were then vacuum-filtered through polycarbonate or PTFE filters, respectively, of 0.4 μm pore size. After drying with hot air, CNT films were peeled off from the filtration membrane (“dense” processing). Alternatively, to succeed in a more foamy structure of the paper, vacuum filtering was stopped, while some solvent was remaining, and then the drying was performed (“foamy” processing). By this, the nanotubes were able to arrange with each other in longer distances, because of the absence of vacuum pressure. The average thickness of the oxidized buckypapers was approximately 100 μm and their diameter about 7 cm. For the epoxidized buckypapers, the average thickness of the epoxidized buckypapers was approximately 170 μm for the water treated and 230 for the dichloromethane treated. Dichloromethane was also tested with oxidized nanotubes, but they were not able to produce a bulk structure. Also, the alternative method for a more foamy structure didn’t work for the epoxidized nanotubes. Table 4 shows the different types of buckypapers tested.

**Table 4:** Types of buckypapers tested.

Chemical treatment	Solvent	Drying	Structural integrity	Sample name
Oxidation	Water	“Dense” processing	Yes	Oxidized-dense
		“Foamy” processing	Yes	Oxidized-foamy
	Dichloromethane	“Dense” processing	No	–
		“Foamy” processing	No	–
Epoxidation	Water	“Dense” processing	Yes	Epoxidized-H_2_O
		“Foamy” processing	No	–
	Dichloromethane	“Dense” processing	Yes	Epoxidized-CH_2_Cl_2_
		“Foamy” processing	No	–

To examine how successful the functionalization of the nanotubes was, thermogravimetric analysis (TGA) was performed. A quantity of CNTs was heated at 700 °C in nitrogen atmosphere, by 10 °C/min. The analysis was carried out with a TA Q50 instrument.

The textural characteristics (porosity) of the neat buckypapers were examined by mercury porosimetry analysis. Mercury intrusion curves of the studied CNT sheets were obtained using a QuantachromePoreMaster 60 Hg Porosimeter. The capillary pressure, *P*_c_, has been replaced by the diameter of an equivalent cylindrical tube, *D*, according to the relation:

*P_c_* = 4*γ* cos*θ*/ *D* (1)

where *γ* is the surface tension of Hg (0.48 N m^−1^) and *θ* is the contact angle (40°).

SEM photos from the cross section of the buckypapers were taken in order to examine the structure of the nanotubes. We used a LEO SUPRA 35 VP scanning electron microscope.

Mechanical testing of the neat buckypapers was performed in a TA Instruments Dynamic Mechanical Analyzer Q800 with a displacement rate of 500 μm min^−1^ on strips of the dimensions 30 mm × 4 mm. For each film type, stress-strain curves were measured for 5 strips.

Finally, conductivity characteristics of the buckypapers were obtained with a conventional four probe method [42].

## 4. Conclusions

In this work, different types of buckypapers were fabricated. It was found that the oxidized nanotubes produce a very dense paper, with very small pores. In addition, epoxidized nanotubes create a foamier buckypaper, with larger pore sizes. The mechanical and electrical properties of the buckypapers strongly correlate with their structure: the denser the paper is, the higher the modulus and electrical conductivity is. These findings give a quite useful understanding of the structure-properties relation of the buckypapers, for potential utilization in many applications, such as reinforcing agents in polymer composites.
